# Small molecule CXCR4 antagonists block the HIV-1 Nef/CXCR4 axis and selectively initiate the apoptotic program in breast cancer cells

**DOI:** 10.18632/oncotarget.24580

**Published:** 2018-02-26

**Authors:** Ming-Bo Huang, Kyle E. Giesler, Brooke M. Katzman, Anthony R. Prosser, Valarie Truax, Dennis C. Liotta, Lawrence J. Wilson, Vincent C. Bond

**Affiliations:** ^1^ Department of Microbiology, Biochemistry and Immunology, Morehouse School of Medicine, Atlanta, Georgia 30310, United States; ^2^ Department of Chemistry, Emory University, Atlanta, Georgia 30322, United States

**Keywords:** CXCR4 compounds, breast cancer cell lines, selective targeting apoptosis, depolarization, NefM1

## Abstract

The chemokine receptor CXCR4 plays an integral role in the development of highly metastatic breast cancer and in the pathogenesis of chronic HIV infection. In this study, we compared the effects of CXCR4 antagonists on apoptosis induction in hematopoietic cells and in tumor cells. We incubated cells expressing CXCR4 with a series of CXCR4 antagonists and subsequently exposed the cultures to a pro-apoptotic peptide derived from the HIV-1 Nef protein (NefM1). The NefM1 peptide contains residues 50–60 of Nef and was previously shown to be the sequence necessary for Nef to initiate the apoptotic program through CXCR4 signaling. We found that several of the compounds studied potently blocked Nef-induced apoptosis in Jurkat T-lymphocyte cells. Interestingly, many of the same compounds selectively triggered apoptosis in MDA-MB-231 breast cancer cells, in some cases at sub-nanomolar concentrations. None of the compounds were toxic to lymphocyte, monocyte or macrophage cells, suggesting that aggressive breast cancer carcinomas may be selectively targeted and eliminated using CXCR4-based therapies without additional cytotoxic agents. Our results also demonstrate that not all CXCR4 antagonists are alike and that the observed anti-Nef and pro-apoptotic effects are chemically tunable. Collectively, these findings suggest our CXCR4 antagonists have promising clinical utility for HIV or breast cancer therapies as well as being useful probes to examine the link between CXCR4 and apoptosis.

## INTRODUCTION

CXCR4 is a G-protein coupled receptor charged with a multitude of biological functions including T-cell activation [1], chemotaxis [2], vascularization [3], and cellular proliferation [4]. The receptor features seven α-helices organized in a barrel-like array embedded within the plasma membrane in a trans-membrane configuration in order to mediate signaling events between the extracellular and intracellular milieu. Signaling via CXCR4 is initiated upon binding to a sole ligand, CXCL12, which triggers a series of intimate conformational changes that channel through the receptor to G-coupled proteins that reside near the inner leaflet of the cell membrane [5]. In contrast to other chemokine receptors which may bind several ligands, CXCR4 is unique in that its signaling pathway cannot be activated by chemokines other than CXCL12. Consequently, CXCR4 has a specialized physiological role that has been appreciated in neonatal development [6], brain development [7], and hematopoiesis [8] amongst others.

CXCR4 also promotes neoplastic transformations in various cell types to facilitate cellular proliferation, invasiveness, and survival that results in poor prognosis and enhanced morbidity [9]. It also plays a prominent role in HIV-1 infection and interacts with several HIV-1 viral proteins including gp120, Tat, and Nef [10–13]. Previous studies in our laboratory have shown that HIV-1 Nef protein is present in the plasma of HIV-1 infected patients in amounts sufficient to induce apoptosis; that the apoptotic activity of the HIV-1 Nef protein can be localized in two 10-amino acid regions called Motif 1 (M1) and Motif 2 (M2) and that the extracellular Nef protein targets CD4^+^ T lymphocytes for apoptosis by interacting with the chemokine receptor CXCR4 [14–17]. The gp120/CXCR4 axis mediates viral entry and the Nef/CXCR4 axis triggers apoptosis in uninfected bystander T cells [18, 15, 16]. The interaction of Nef with CXCR4 may contribute to T-cell depletion and the immune dysfunction that characterizes AIDS. CXCR4 antagonists are known to block entry of CXCR4-tropic HIV-1 viruses into cells, however, it remains unclear if these compounds also have the capacity to curb Nef-induced apoptosis.

The chemotactic properties of CXCR4 combined with its ability to activate pro-survival mechanisms and stimulate cellular proliferation places the CXCR4/CXCL12 axis at the vanguard of metastasis and tumorigenesis for many cancers [19–20]. Indeed, the over-expression of CXCR4 has been linked to over 20 different types of cancer and is associated with advanced metastatic disease and a poor clinical outcome. Consequently, CXCR4 has garnered significant interest as a therapeutic target and small-molecule CXCR4 antagonists have emerged as attractive agents for the treatment of several carcinomas, leukemias, and lymphomas [19]. CXCR4 antagonists may prevent CXCL12 binding to CXCR4 to compromise the activation of downstream signaling pathways required for proliferation, angiogenesis, and chemotaxis which, in turn, sensitizes neoplastic cells to cytotoxic agents. AMD3100 is a clinically-approved CXCR4 antagonist used in combination with granulocyte-colony stimulating factor (G-CSF) to mobilize leukemia and lymphoma cells into the bloodstream where they become vulnerable to cytarabine and anthracycline chemotherapy [21–23].

With respect to HIV infection, it has been appreciated for some time that HIV exploits CXCR4 as a co-receptor for viral entry, via interaction with the viral surface protein, Env [24]. However, other HIV proteins are also known to interact with CXCR4 including Nef, gp120, and Tat [14, 25, 26]. Several converging lines of evidence have identified Nef as a key player in the progression to AIDS which sparked our interest in the development of potential anti-Nef therapeutic agents. Nef is a small, *N*-myristoylated viral adapter protein that was shown to modulate the presentation of various immune receptors at the plasma membrane in order to evade and counter the host immune response [27–28]. The immunomodulatory properties of Nef are likely not limited to the confines of a given cell type and it has been demonstrated that Nef expression in transgenic mice models is associated with rapid CD4^+^ T cell decline and the onset of a characteristic AIDS-like disease in the complete absence of virion production [29–30].

We previously reported that the full length Nef protein and Nef peptides containing residues 50–60 (herein referred to as motif 1, M1) bind to CXCR4 and initiate the apoptotic program in CD4^+^ T cells which may contribute to the pathogenesis of AIDS *in vivo* [14, 15]. We then proceeded to exploit the apoptotic kinship between Nef M1 and CXCR4 to suppress the growth and metastasis of primary colorectal tumors in mice [31–32] and recently found that M1 exhibits profound anti-proliferative activity against various CXCR4-expressing breast carcinomas [33–34]. M1's ability to eliminate cells is advantageous for the treatment of cancer, however, this effect is non-selective and also eliminates physiologically relevant cells such as PBMCs and other immune cells which HIV exploits to destroy the host immune system. Consequently, the utilization of Nef (or M1) as an anti-cancer regimen may result in indiscriminate apoptosis and myelosuppression during several rounds of chemotherapy. Herein, we report that a series of small molecule CXCR4 antagonists can selectively induce apoptosis in MDA-MB-231 breast cancer cells at sub-nanomolar concentrations. Importantly, none of the compounds studied impacted the viability of Jurkat T-lymphocyte cells but rather protected these cells from apoptosis when the cultures were co-incubated with M1. Our results support a vast body of literature that validates CXCR4 as a promising target for cancer therapy and demonstrate that small-molecule CXCR4 antagonists have novel therapeutic potential for HIV infection beyond their activity against viral entry by blocking Nef induced T-cell depletion.

## RESULTS

### Selection and biological characterization of active CXCR4 antagonists

**Figure 1:**
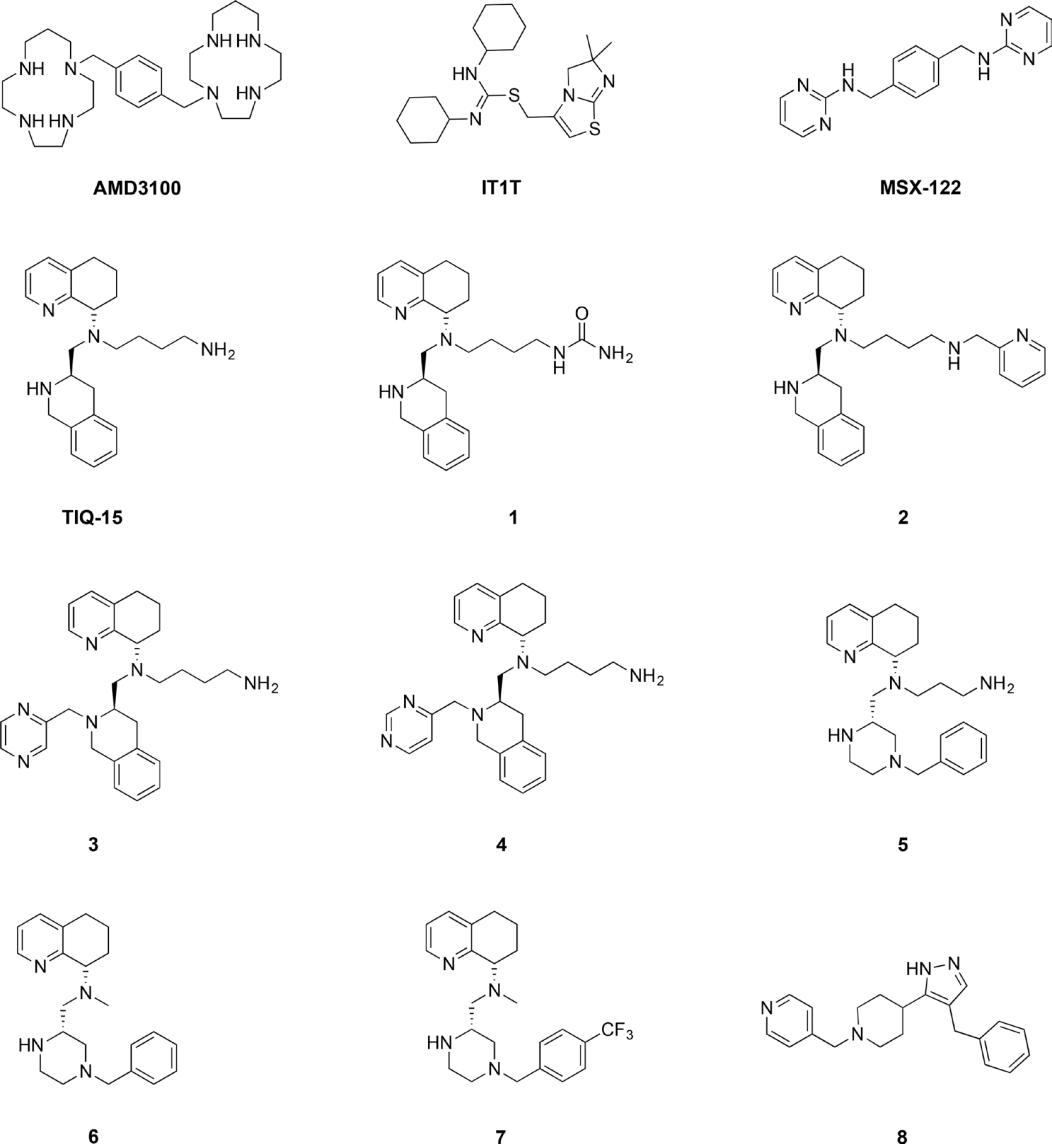
Structures of CXCR4 antagonists used in this study

We recently described two series of CXCR4 antagonists and characterized their interaction with CXCR4, including their ability to antagonize HIV viral entry [35, 36]. We also previously discovered a series of dual CCR5/CXCR4 entry inhibitors with unique non-nucleoside reverse transcriptase (NNRTI) activity against HIV [37]. From these works, we selected a handful of compounds that exhibit varying degrees of CXCR4 antagonism and included them in the present study (Figure 1). We also included the known antagonists AMD3100, MSX-122, IT1t and TIQ-15, as well as tetrahydroisoquinoline (THIQ) compounds (1-4), piperazine (PIP) compounds (5-7) and pyrrolo-piperidine compound 8 (Figure 1) [35–39]. Prior to screening in both Jurkat and breast cancer cells, two assays were used to characterize their interaction with CXCR4: (i) CXCL12 induced calcium flux; and (ii) the HIV-1_IIIB_ MAGI entry assay (Table 1). From these assays, the compounds in Figure 1 can be grouped into four major classes; (i) compounds that block HIV entry with similar therapeutic efficacies to SDF-1 (IT1t, TIQ-15, 3, 5, 6), (ii) compounds that have selectivity towards blocking HIV entry over CXCR4 antagonism (AMD3100, 4, 7, 8), (iii) compounds that have selectivity towards CXCR4 antagonism over HIV entry (1, 2), and (iv) one compound that has poor responses in both assays (MSX-122) but has been shown to have some type of CXCR4 interaction by other methods. CXCR4-mediated HIV entry was abrogated at sub-micromolar concentrations in HeLa cells (MAGI assay) for all compounds except 7 and MSX-122. Collectively, these data suggest the compounds in Figure 1 antagonize CXCR4 with varying affinities which likely reflect different binding modes to the receptor. This range in activity is useful for probing signaling transduction pathways mediated by CXCR4 and provides us with a broad set of tools to study the impact of CXCR4 antagonism against different ligands (such as Nef M1 and CXCL12) in various cell types.

**Table 1:** Biological characterization of CXCR4 antagonists

Compound	Jurkat M1 Inhibition EC_50_ (μM)	HeLa HIV-1_IIIB_ MAGI IC_50_ (μM)	CEM SDF-1 Ca^2+^ Flux IC_50_ (μM)	MDA-MB-231 Depolarization EC_50_ (μM)
AMD3100	0.470	0.002	0.593^*^	0.123
It1t	0.0007	0.01	0.008	0.00625
MSX-122	12.0	>10	>10	0.081
TIQ-15	0.0007	0.005	0.003	0.00054
1	0.0003	0.21	0.004	0.0008
2	0.007	0.12	0.001	0.00037
3	0.0035	0.79	0.11	0.015^**^
4	0.0515	0.003	7.7	>100^***^
5	0.010	0.03	0.19	0.00039
6	0.0533	0.85	3.3	10.0^**^
7	12.7	8.01	>50	85
8	1.10	0.78	>50	0.028

^*^ Literature value.

^**^ Partial or incomplete inhibition – Did not obtain > 65% inhibition at 100 μM.

^***^ Incomplete inhibition, ~20% at 100 μM.

### CXCR4 antagonism protects jurkat T-lymphocytes from M1-induced apoptosis

**Figure 2:**
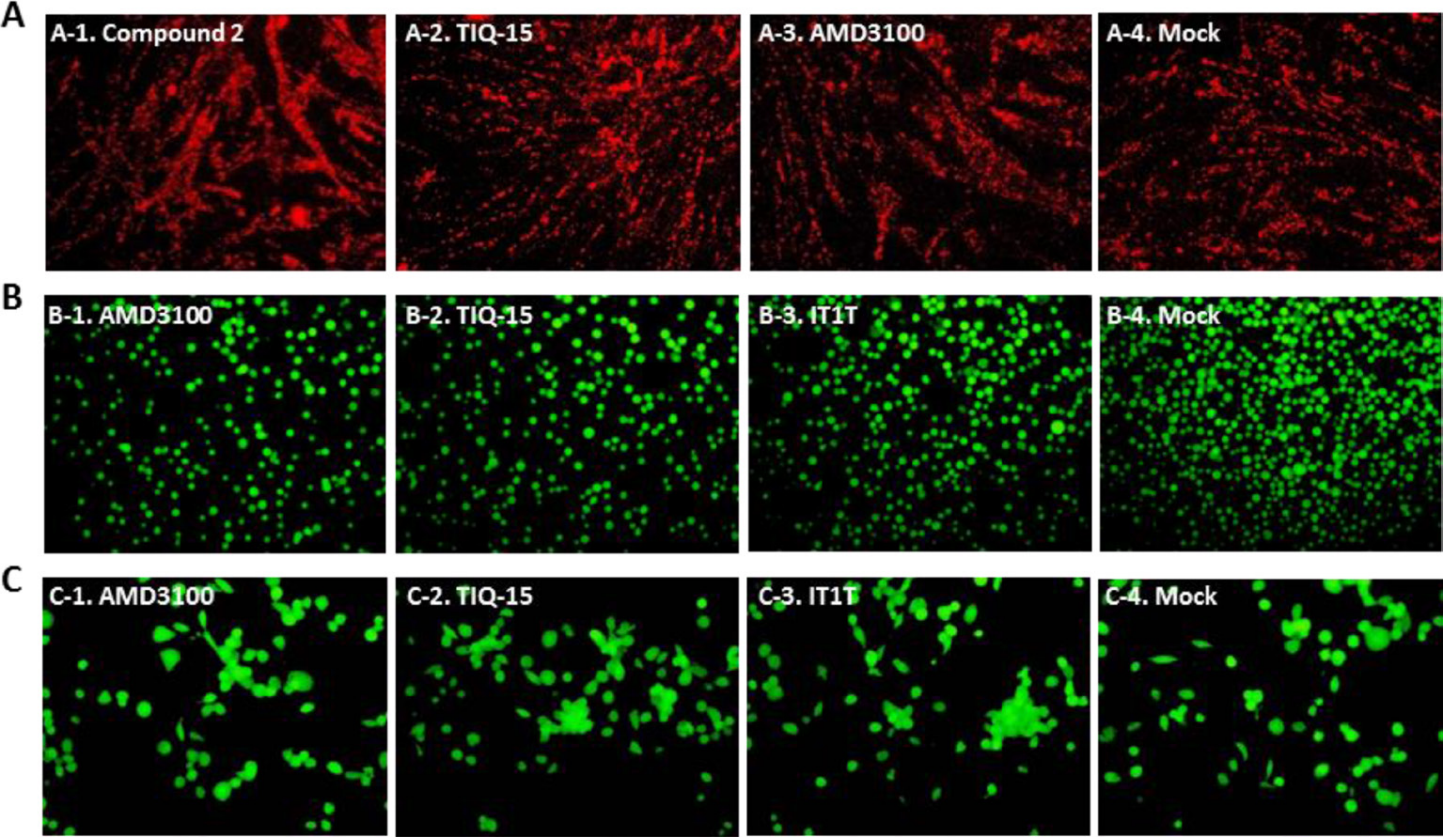
Absence of CXCR4 inhibitor compounds toxicity on HUVEC, jurkat and THP-1 macrophage as determined by either JC-1 stain assay or FD/PI assay Several cell types were treated with either 1000 nM AMD3100, or 50 nM TIQ-15, IT1T, or Compound 2 for 24 hours. (**A**) HUVEC were assayed via JC-1; (**B**) Jurkat cells, and (**C**) THP-1 Macrophage were assayed via FD/PI. The images were taken via fluorescence microscopy and arranged with Adobe Photoshop 6.0 software.

**Figure 3:**
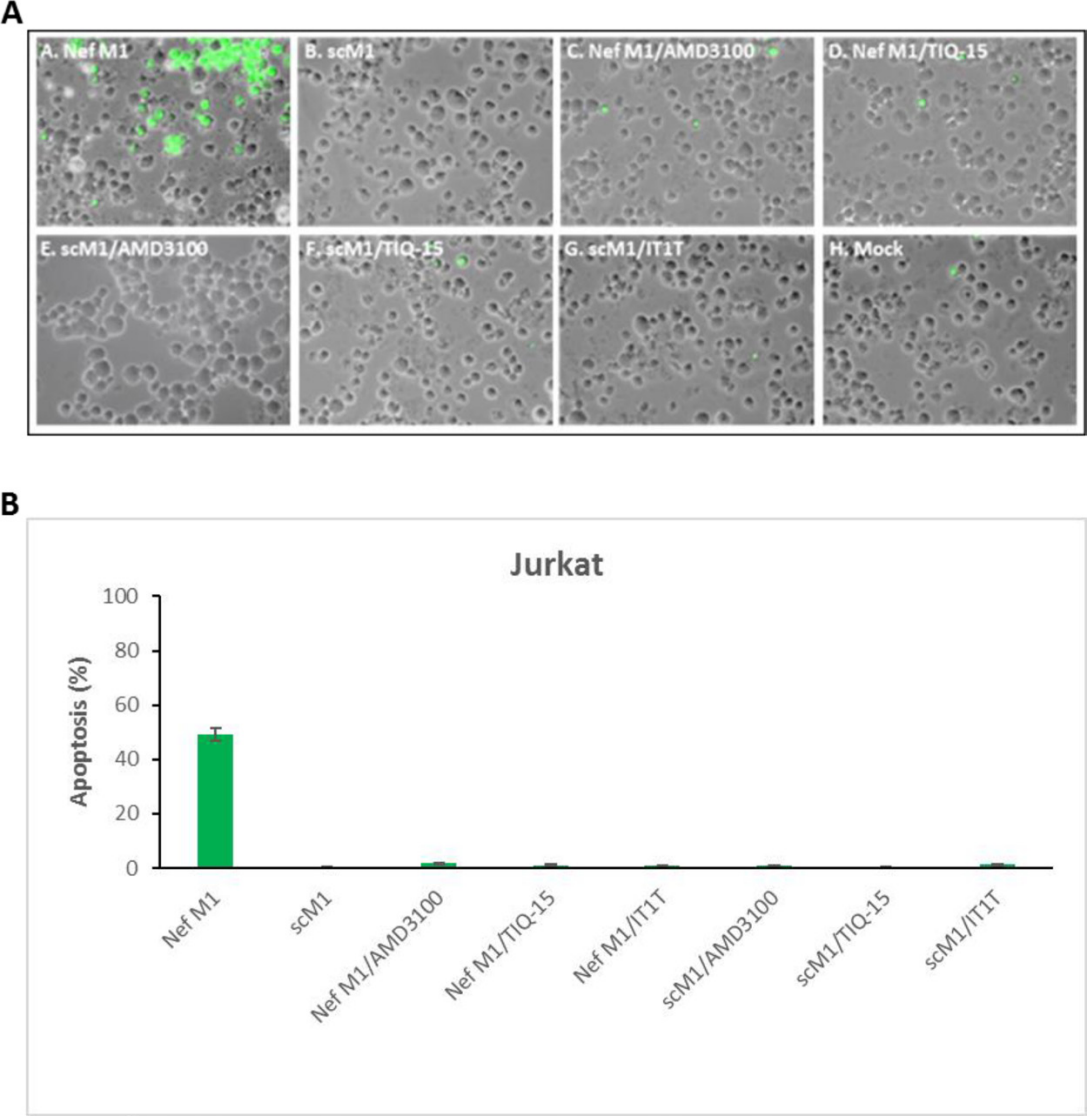
CXCR4 antagonists inhibited M1 peptide-induced apoptosis in jurkat cultures Jurkat cells were treated for 24 hours with 10 nM of either HIV-1 Nef M1peptide or 10 nM scM1(sc = scambled), or with M1 or scM1 and 1000 nM AMD3100, or 50nM TIQ-15, or 50 nM IT1T. (**A**) Fluorescent microscopy images of Jurkat cultures stained via TUNEL. (**B**) TUNEL stained cells were counted and quantified. Fluorescent microscopy images were arranged with Adobe photoshop 6.0 software and analyzed by Image-Pro 6.3 software (Media Cybernetics, Silver Spring, MD).

Our initial aim was to demonstrate that CXCR4 antagonism confers protection against Nef-mediated apoptosis. We initially incubated Jurkat T-lymphocytes with selected compounds (AMD3100, TIQ-15 and IT1t) to show the lack of any toxicity of these molecules (Figure 2B). Subsequently, Jurkat cultures exposed to 10 ng/mL of the M1 peptide (TNAACAWLEAQ) in the absence of antagonist, displayed apoptosis (Figure 3) after 24 h of exposure. When the cultures were incubated with a control, scrambled M1 peptide (scM1, ALAETCQNAWA), no apoptosis induction was observed confirming that the activity of M1 is sequence dependent (Figure 3). As expected, prophylactic exposure of Jurkat cells to 50 nM of the known CXCR4 antagonist IT1t followed by incubation with M1 conferred complete protection from M1-mediated apoptosis (Figure 3). We discovered that this effect was dose dependent and that 7 nM of IT1t was sufficient to prevent 50% of the apoptosis caused by M1 when determined by JC-1 staining (Table 1, EC_50_= 0.7 nM). AMD3100 also curbed M1-induced apoptosis albeit at a 500-fold higher concentration (EC_50_ = 470 nM). These results demonstrate that IT1t and AMD3100 have the capacity to protect Jurkat cells from M1-mediated apoptosis and prompted us to evaluate the activity of the remaining compounds in Figure 1. The data for all compounds is is presented in Table 1. Also included is the concentration of each compound required to inhibit M1-induced mitochondrial depolarization by 50% (EC_50_). Notably, several compounds from our THIQ series potently inhibited M1-mediated depolarization in Jurkat cells at low nanomolar or sub-nanomolar concentrations which are therapeutically accessible. In particular, AMD3100, IT1t TIQ-15 and compound 2 were non-toxic to Jurkat cells, HUVEC and THP-1 macrophages and failed to effect mitochondrial depolarization for three examined via JC-1 (Figure 2A–2C). As controls, the CCR5 antagonist Maraviroc (MVC) and the nucleoside reverse transcriptase inhibitor AZT did not confer protection against M1, and Jurkat cells incubated with MVC + M1 showed similar levels of depolarization as cells incubated with M1 alone (Data not shown).

### CXCR4 antagonism induces apoptosis in MDA-MB-231 breast cancer cells

**Figure 4:**
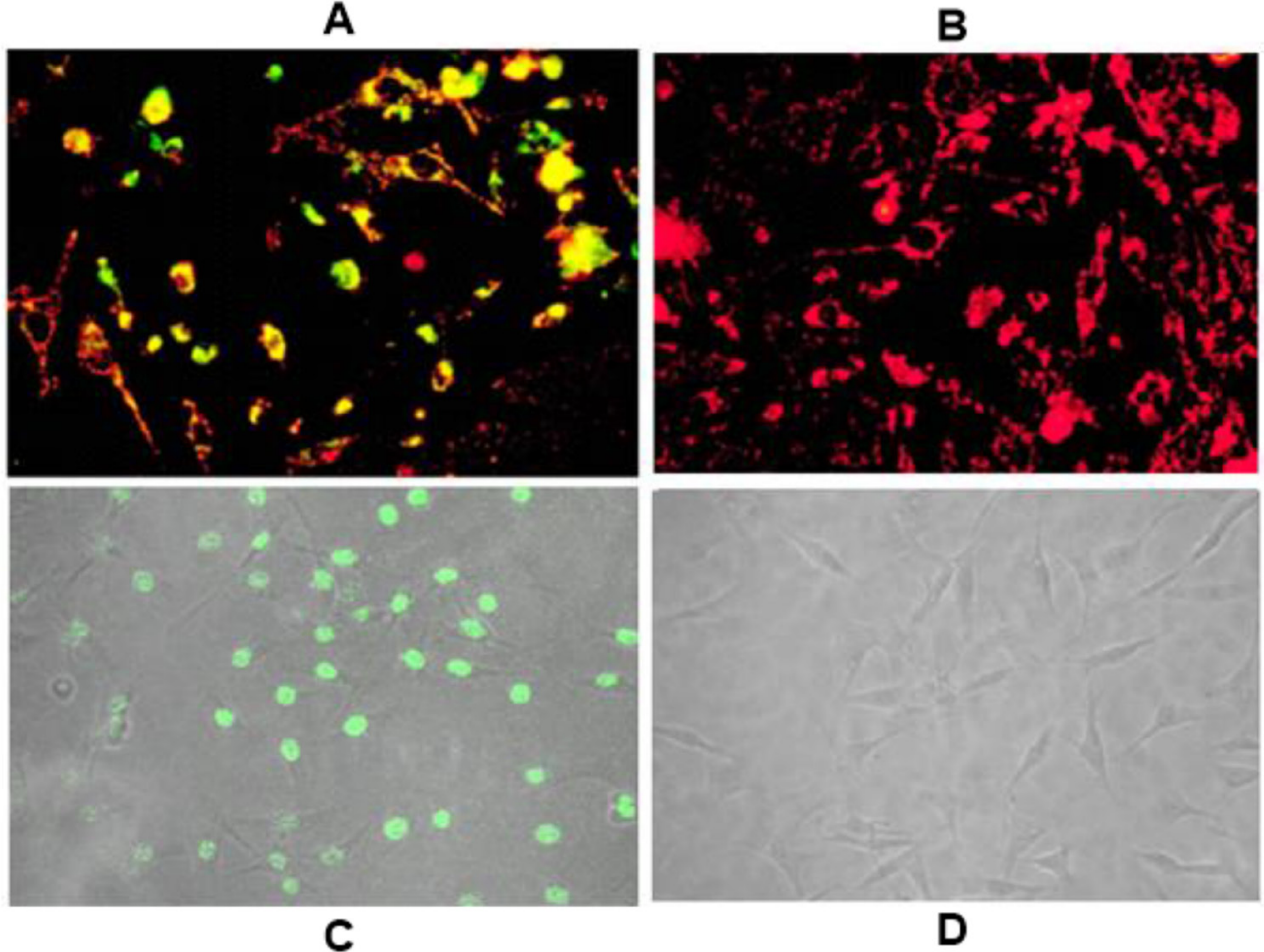
Effect of TIQ-15 on MDA-MB-231 breast tumor cells (**A**) MDA-MB-231 cells were treated with 7 nM of TIQ-15 for 24 h then stained with JC-1. Substantial (>90%) mitochondrial depolarization was observed (yellow) (**B**) MDA-MB-231 cultures were mock treated and stained with JC-1 (red). (**C**) MDA-MB-231 cells treated with 7 nM of TIQ-15 for 24 h then analyzed by TUNEL. Significant apoptosis was observed (green). (**D**) MDA-MB-231 cells mock treated were analyzed via TUNEL. No apoptosis was observed. The images were taken via fluorescence microscopy and arranged with Adobe photoshop 6.0 software.

To determine if CXCR4 antagonism prevents M1-induced apoptosis in other cell types, we chose to work with the breast cancer cell line MDA-MB-231 which over-expresses CXCR4 [32]. We previously reported that M1 exhibits profound anti-proliferative activity against MDA-MB-231 breast cancer cells [33–34] and posited that CXCR4 antagonism would protect these cells from M1. However, when we exposed MDA-MB-231 cells to 10 ng/mL of M1 and the compound, we still observed apoptosis (data not shown). Thus, we repeated this experiment without M1 treatment exposing MDA-MB-231 cells to the antagonist compound TIQ-15 alone for 24 hours and then assayed via either JC-1(Figure 4A) or TUNEL (Figure 4C). Considerable depolarization (Figure 4A and 4B) and TUNEL (Figure 4C and 4D) staining was observed in both cases with the observed amounts of depolarization or TUNEL labeling being similar. This unexpected result suggested a differential effect in cancer cells versus other cell types.

### CXCR4 gene and surface protein expression was observed in all cell lines examined except MDA-MB-468

**Figure 5:**
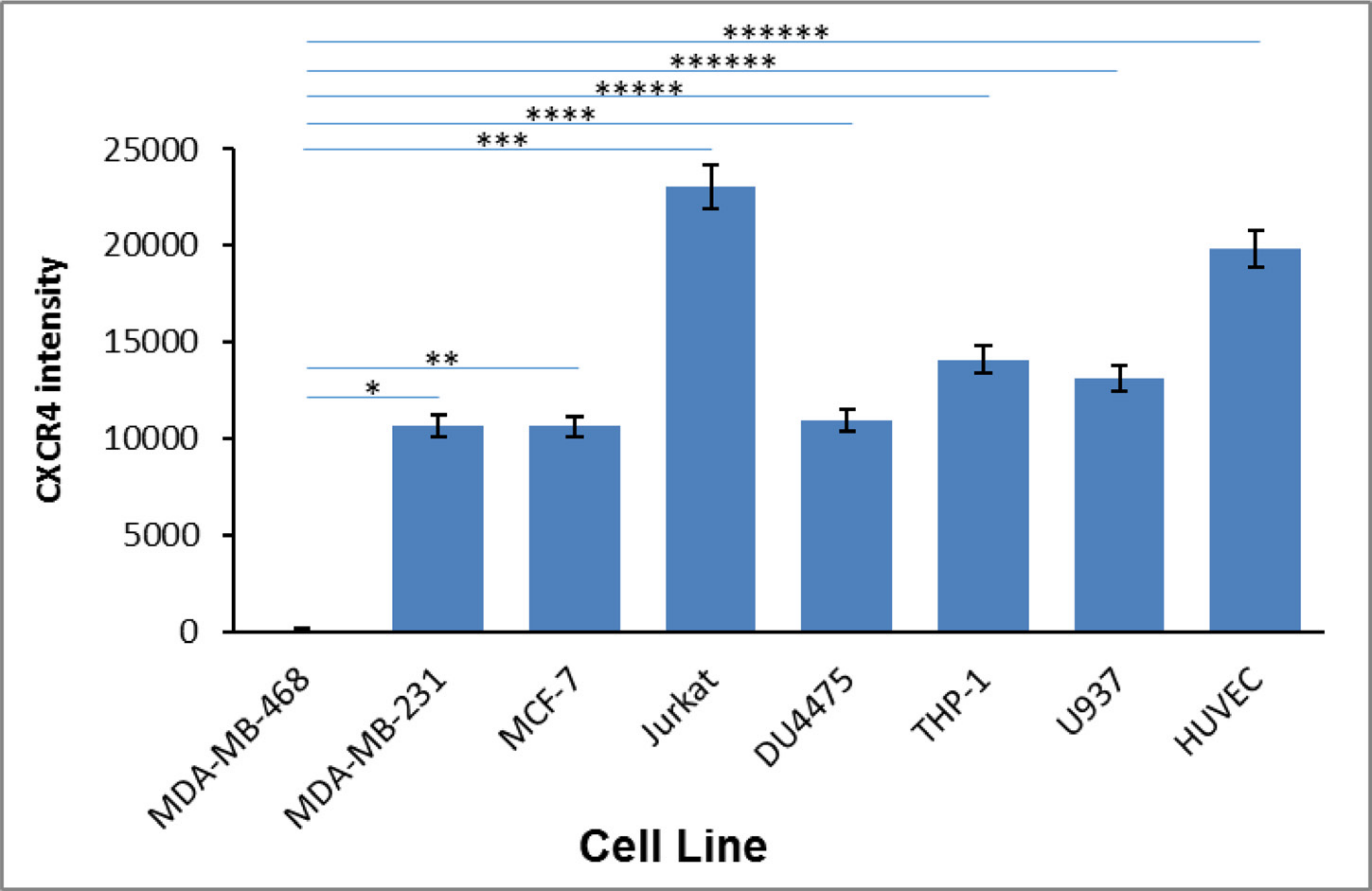
CXCR4 mRNA expression in various cell types The expression of CXCR4 mRNA was determined via RT-PCR in breast tumor lines, MDA-MB-468 and MDA-MB-231, MCF-7, DU4475, and in HUVEC primary cells, THP-1 and U937 monocytes, and Jurkat lymphocytes. Asterisks (^*^) indicate significant differences (*p* < 0.05) relative to control MDAMB-468: ^*^p < 4.5E-08, ^**^p < 4.2E-08, ^***^p < 1.7E-13, ^****^p < 6.3E-08, and ^*****^p < 3.8E-6, ^******^p < 4.5E-16.

**Figure 6:**
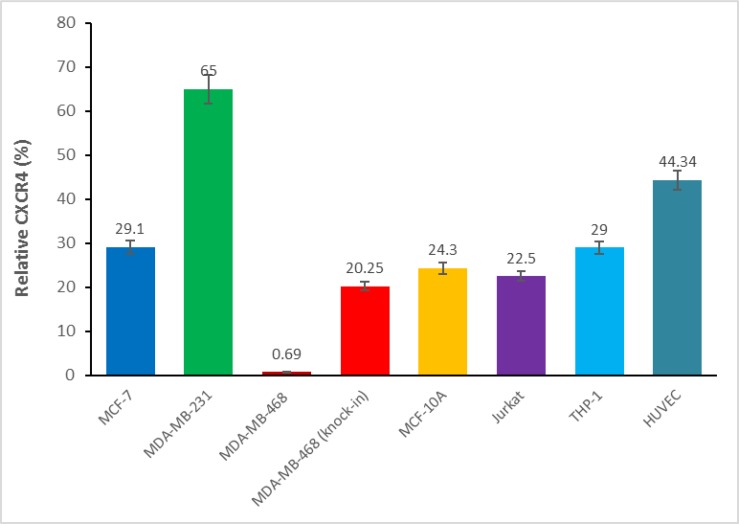
Cell surface CXCR4 expression in various cell types The cell surface CXCR4 expression was determined via flow cytometry in MDF-7, MDA-MB-231, MDA-MB-468, MDA-MB-468 (knock-in CXCR4) breast cancer cells, non-tumorigenic MCF-10A cells, HUVEC primary endothelial cells, THP-1 monocytes and Jurkat lymphocytes.

To confirm CXCR4 expression, first we screened for CXCR4 expression in our breast carcinoma cells lines MDA-MB-468, MDA-MB-231, MCF-7, DU4475, as well as in THP-1, HUVEC, U937, and Jurkat through RT-PCR analysis (Figure 5) Total RNA was prepared and subjected to RT-PCR analysis to detect CXCR4-specific mRNA. As expected, all cells examined displayed expression of CXCR4 mRNA, except MDA-MB-468 cells (which are known to not express CXCR4). Second, we further confirmed that all cell lines that showed positive CXCR4 mRNA also showed CXCR4 receptor expression on the cell surface. All cell lines used in our study were analyzed for CXCR4 surface receptors by florescence via Flow Cytometry with a tagged antibody (Figure 6). With the exception of the MDA-MB-468 CXCR4 negative breast cancer cells, all other cell types (Jurkats, THP-1s, HUVECs, breast cancer cells (MCF-7, MCF-10A, MDA-MB-231 and 468 “CXCR4 knock ins”) had slight variations in surface receptor expression (within 3-fold). Thus, cells used in our study had validated CXCR4 presence adding to our confidence in this protein playing a role in the pharmacology observed in our study.

### Mitochondrial depolarization was induced by the CXCR4 antagonists

**Figure 7:**
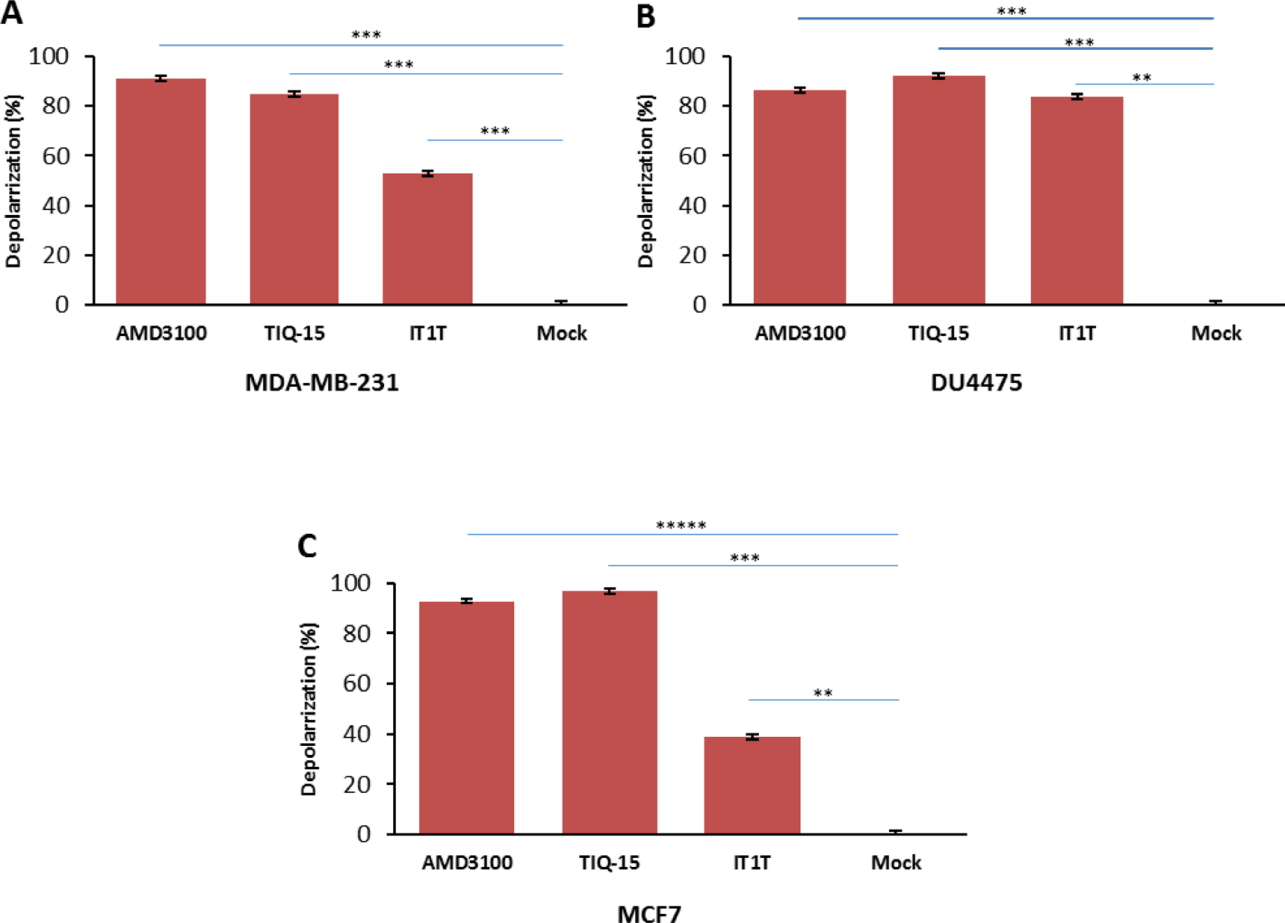
Effect of small molecule CXCR4 antagonists on breast cancer cells Three breast cancer cell lines were treated with either: 123 nM AMD3100, 0.54 nM of TIQ-15, or 6.25 nM of IT1T for 24 hours. Mitochondrial depolarization was analyzed via JC-1 stain assay. (**A**) bar graphs showing depolarization levels of MDA-MB-231 cells. Error bars represent the mean ± SD of two independent experiments. Asterisks (^*^) indicate significant differences (*p* < 0.05) relative to control ^***^p < 0.0004. (**B**) DU4475 cell depolarization levels were assayed using JC-1 staining assay, ^**^p < 0.001, ^***^p < 0.0003. (**C**) MCF-7 cell depolarization levels were assayed via JC-1 staining, ^**^p < 0.001, ^***^p < 0.0004 and ^*****^p < 0.000002.

**Figure 8:**
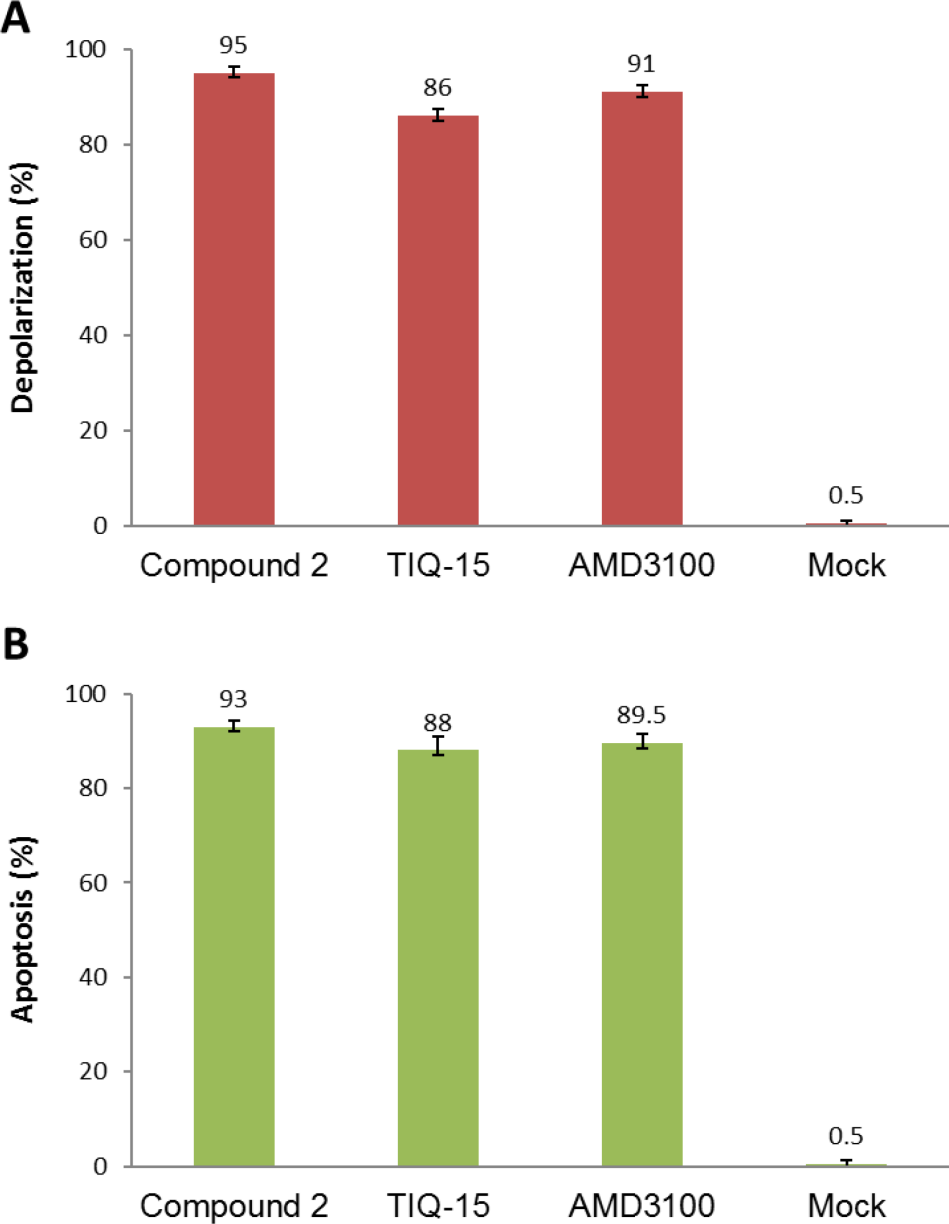
Similarity between depolarization and apoptotic effects of the antagonists (**A**) the top panel shows a depolarization analysis in MDA-MB-231 cells for the thee listed antagonists via JC-1 staining, mock treated cells. (**B**) the bottom panel shows data from a TUNEL assay for the same compounds in the same MDA-MB-231 Breast tumor cells. Error bars represent the mean ± SD of two independent experiments.

We then examined the effects of three of our compounds as well as AMD3100 in three different breast tumor lines (Figure 7). Breast tumor lines MDA-MB-231 (7A), MCF7 (7C), and DU4475 (7B) were treated with either 123 nM AMD3100, 0.54 nM of TIQ-15, or 6.25 nM of IT1T for 24 hours with subsequent analysis for mitochondrial depolarization via JC-1 stain assay. Percent cell depolarization was quantified via microscopic analysis with bar graphs showing depolarization levels. As can be observed, all compounds examined displayed depolarizing effects on all three target breast tumor lines. As discussed above, we had observed similar quantities of depolarization and apoptosis in the MDA-MB-231 cultures with TIQ-15 (Figure 4). We went back to look at depolarization and apoptosis with these other compounds (Figure 8). Breast tumor line MDA-MB-231 cultures were treated with compound 2, TIQ-15, or AMD3100 and then depolarization was quantitated via JC-1 staining (Figure 8A), or apoptosis via TUNEL (Figure 8B). The observed effects were almost identical; suggesting that mitochondrial depolarization and apoptosis are linked via the same postulated CXCR4 based mechanism.

### Induction of depolarization by antagonist Compounds was CXCR4 dependent

**Figure 9:**
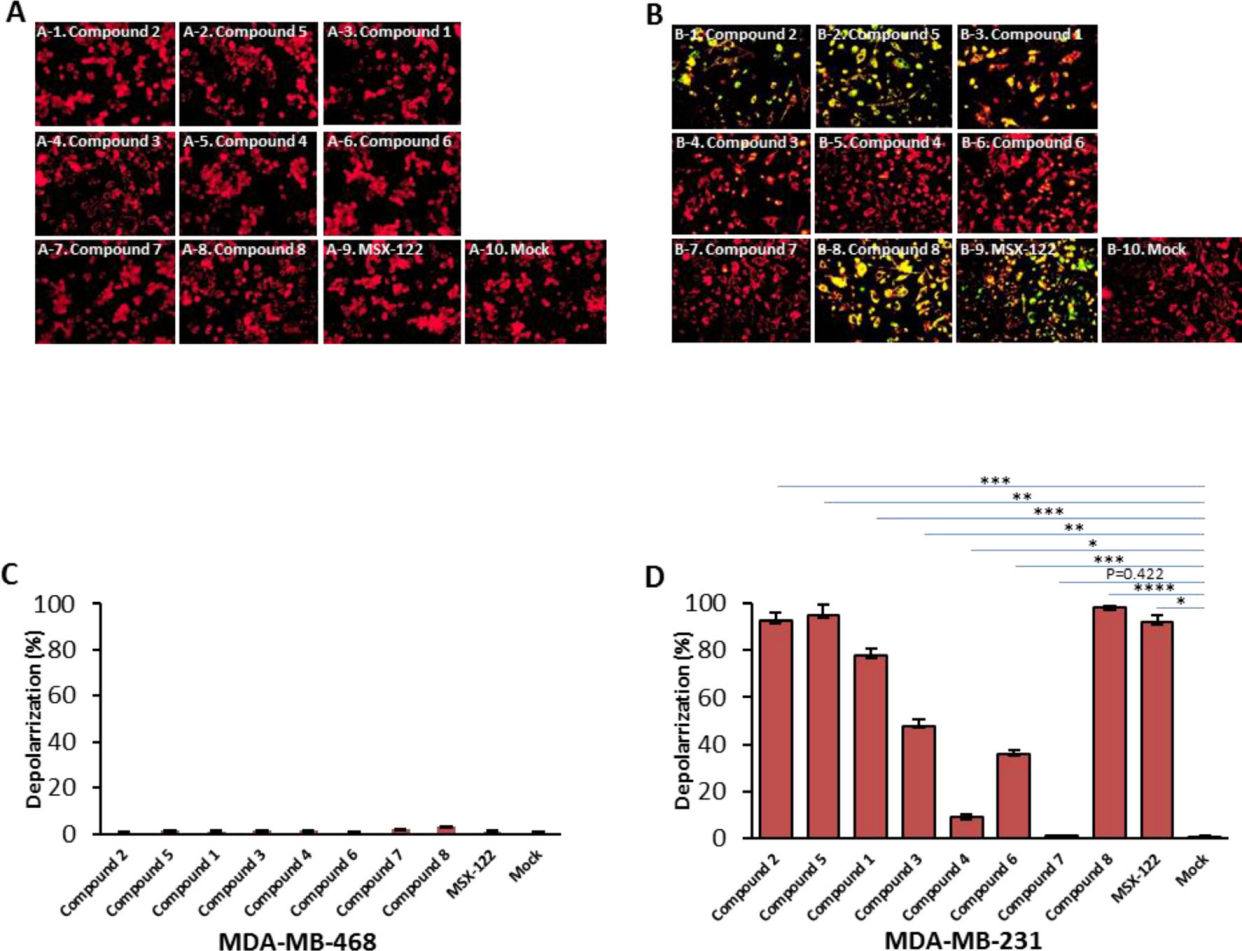
The crucial role of CXCR4 receptor in the small molecule antagonists effect on breast cancer cells Cells were treated with CXCR4 antagonists: 0.37 nM of compound 2, 0.39 nM of compound 5, 0.8 nM of compound 1, 15 nM of compound 3, 100 uM of compound 4, 169 nM of compound 6.85 μM of compound 7, or 28 nM of compound 8 and 81 nM of MSX-122 for 24 hours. The cells were then analyzed for depolarization via JC-1 staining. (**A**) MDA-MB-468 cells which lacks expression of CXCR4. (**B**) MDA-MB-231 cell line that express CXCR4. Images were taken via fluorescence microscopy and arranged with Adobe photoshop 6.0 software; bar graphs quantifying depolarization levels of (**C**) MDA-MB-468 cells; (**D**) MDA-MB-231 cells are shown. Error bars represent the mean ± SD of two independent experiments. Asterisks (^*^) indicate significant differences (*p* < 0.05) relative to each compare ^*^p < 0.01, ^**^p < 0.001, ^***^p < 0.0009 and ^****^p < 0.00005.

**Figure 10:**
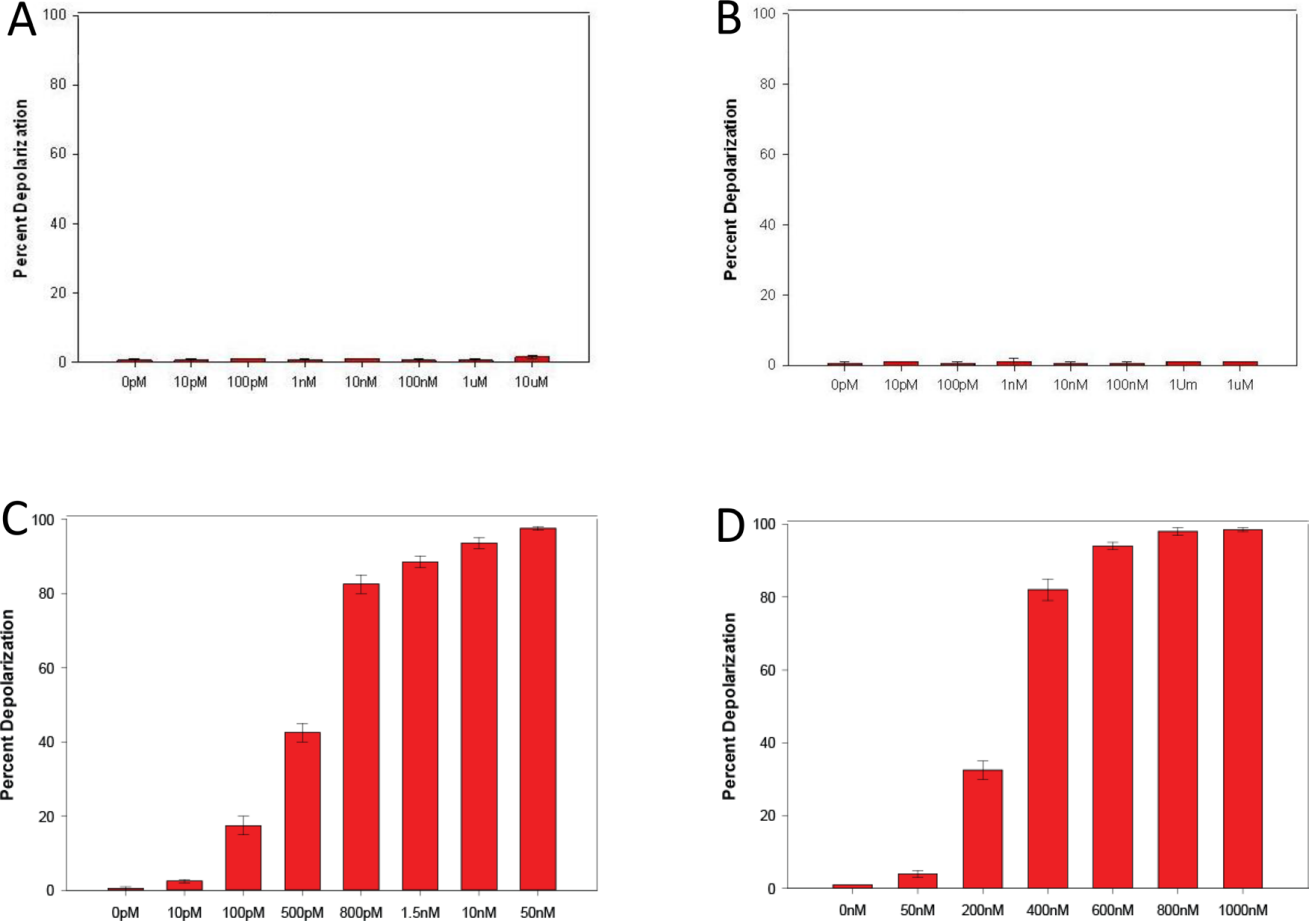
Effects of non-CXCR4 antagonists on MDA-MB-231 breast cancer cells MDA-MB-231 cells were treated via dosage response with AZT (**A** panel) which targets the reverse transcriptase in HIV infected cells, or Maraviroc (**B** panel) which is a CCR5 antagonist positive. Controls are compound TIQ-15 (**C** panel), and AMD3100 (**D** panel). Error bars represent the mean ± SD of two independent experiments.

As mentioned previously, the CXCR4 antagonists in this study were selected based on their differential and ligand-biased properties characterized by the CXCR4 receptor functional calcium flux and HIV-1 entry-based MAGI assays (Table 1). We proceeded to simultaneously assess the potential apoptotic activity of all these compounds (Figure 1), including AMD3100, IT1t, MSX-122, TIQ-15 and compounds 1-8 in MDA-MB-231 cells versus the CXCR4 negative MDA-MB-468 breast cancer cells. TIQ15 was initially assessed and incubated with MDA-MB-231 cells at a concentration of 7 nM (the concentration required to inhibit M1-mediated apoptosis in Jurkat cells by 50%). JC-1 staining and fluorescence microscopy revealed that TIQ15 alone induced mitochondrial depolarization in >90% of the cells (Figure 9B) versus none observed in the 468 cells (Figure 9A). Measuring apoptosis via TUNEL yielded similar results, indicating that the MDA-MB-231 cell line experiences substantial apoptotic induction upon exposure to TIQ15 in the complete absence of M1 peptide (Figure 8B). A dose-response curve was generated, and it was determined that 50% of the cells experienced apoptosis in the presence of 0.37 nM of TIQ-15 (Figure 10C). No depolarization was observed when MDA-MB-231 cells were exposed to AZT (Figure 10A) which targets the reverse transcriptase in HIV infected cells or Maraviroc (MVC; Figure 10B) which is a CCR5 antagonist. Both of these molecules lack affinity for CXCR4. Alternatively, significant depolarization was observed when MDA-MB-231 cultures were exposed to TIQ-15 (Figure 10C) and AMD3100 (Figure 10D), which are CXCR4 antagonists.

To examine this phenomenon further, JC-1 dose-response curves were generated for the remaining compounds in Figure 1 for their ability to induce apoptosis in MDA-MB-231 cells (Figure 9D) and 468 cells (Figure 9C) and their EC_50_ values were calculated (shown in Table 1). Compounds 1-3, 5, TIQ-15 and IT1t elicited activities at low or sub-nanomolar concentrations and four of these (TIQ-15, 1, 2, 5) emerged as our most potent candidates with EC_50_ values in the 370–800 pM range. AMD3100 was about 2000-fold less active than these four compounds with an EC_50_ = 123 nM. Compound 7 was the least active compound identified by this assay (EC_50_ = 85 μM) and also demonstrated similar dismal activity against M1-mediated apoptosis in Jurkat cells (EC_50_ = 12.7 μM). The other compound of note is Compound 4 which showed no apoptosis in MDA-MB-231 cells up to 100 M, but potently blocked M1 induced apoptosis in Jurkat cells (EC_50_ = 52 nM). Taken together, it is clear that CXCR4 antagonism is sufficient to selectively depolarize MDA-MB-231 cells in the absence of M1. None of the compounds showed any apoptotic effects in the CXCR4 negative MDA-MB-468 cells via JC-1 staining (Figures 9A, 9C).

**Figure 11:**
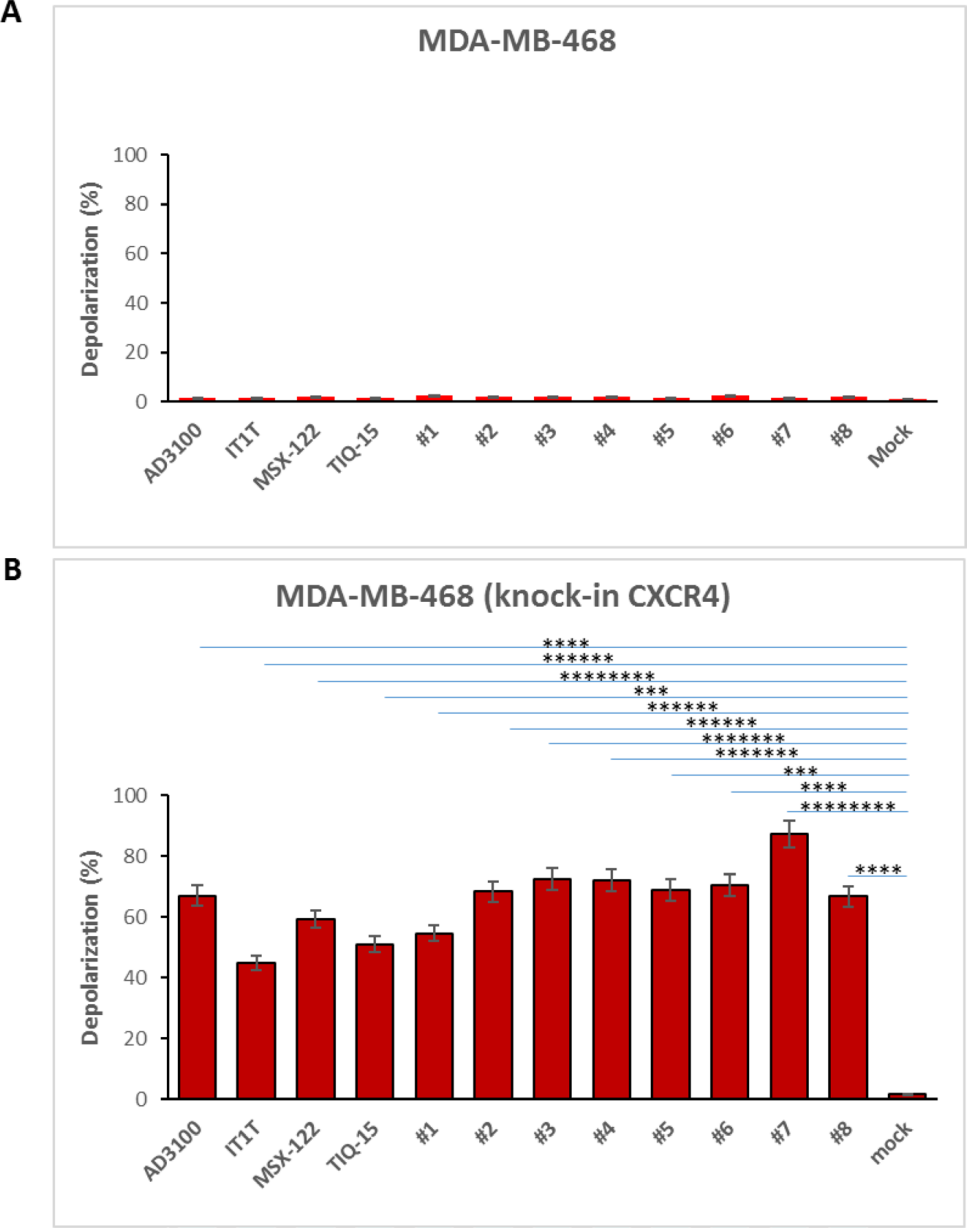
CXCR4 compounds induced depolarization on MDA-MB-468 knock-in CXCR4 cells but not non-CXCR4 MDA-MB-468 cells MDA-MB-468 cells were transfected with 2.5 μg of pc DNA3.1/Zeo(+) CXCR4 via electroporation. Cells were treated with CXCR4 compounds either MDA-MB-468 knock-in CXCR4 cells or non-CXCR4 MDA-MB-468 cells at 37^°^ C for 24 hours and following by JC-1 satin. Depolarization was detected by Fluoresce Microscopy, (**A**) non-CXCR4 MDA-MB-468 and (**B**) MDA-MB-468 knock-in CXCR4 cells in depolarization. Results of two independent experiments are shown. Significant difference relative to untreated control are follows: ^****^p < 1.00E-05, for AMD3100, ^******^p < 1.00E-07 for IT1T, ^********^p < 1.00E-09 for MSX-122, ^***^p < 1.00E-04 for TIQ-15, ^******^p < 1.00E-07 for compound 1, ^******^p < 1.00E-06 for compound 2, ^*******^p < 1.00E-08 for compound 3, ^*******^p < 1.00E-08 for compound 4, ^***^p < 1.00E-04 for compound 5, ^****^p < 1.00E-05 for compound 6, ^********^p < 1.00E-09 for compound 7, ^****^p < 1.00E-05 for compound 8.

To further confirm the role of the CXCR4 receptor in causing the observed mitochondrial depolarization within the MDA-MB-231 cells, we performed a separate experiment. As the MDA-MB-468 cells are devoid of the receptor and showed no mitochondrial depolarization, we performed a modification of these cells by transfecting them with the CXCR4 gene. The CXCR4 “knock in” MDA-MB-468 cells showed positive surface expression (Figure 6) confirming their new status. These cells were then exposed to the CXCR4 antagonists used in our study (Figure 1, Table 1). The results were that the “knock in” cells (Figure 11B) showed a marked effect when measured in the depolarization assay very similar to what was seen in the MDA-MB-231 cells. Again, no effects were observed in the wild-type 468 cells (Figure 11A) indicating a primary role for the CXCR4 receptor in causing mitochondrial disruption.

### Structure-activity relationships

When reviewing the cumulative assay data in Table 1 several observations can be made regarding the classification of the compounds into activity profiles. In eight of the twelve examples, the Nef M1 EC_50_ values were below 100 nM (1–6, IT1t, TIQ-15). In these cases, this activity correlated with both HIV entry blocking ability in the MAGI assay and SDF-1 based CXCR4 functional responses. This was true for compounds with near equal efficacy against blocking HIV entry and CXCR4 receptor functional response (IT1t, TIQ-15, 3, 5, 6), and for compounds in which there was a larger difference between the two (AMD3100, 1, 2, 4, 7, 8). There were compounds where the ability to block Nef M1 in Jurkat cells and induce apoptosis in MDA-MB-231 cells is of near equal efficacy (<10-fold difference in ratio of EC_50_ values: IT1t, TIQ-15, 1-3, 5, 7 and AMD3100), two which favored Nef M1 activity (4, 6) and those in which favored MDA-MB-231 potency (8 and MSX-122). These various patterns of selectivity seen in the jurkat-NefM1 and breast cancer MB-231 assays can be traced to differing profiles of intrinsic HIV-blocking potency in the MAGI assay and CXCR4 functional response but with some exceptions. The compounds that favored the NefM1-jurkat response (4, 6) had good activity in the MAGI assay (IC_50_ < 100 nM) but a poor CXCR4 functional response in the calcium flux assay. For the compounds of similar efficacy in both these assays (IT1t, TIQ-15, 1-3, 5, 7 and AMD3100), four had similar HIV/MAGI-CXCR4/Calcium flux efficacies (IT1t, TIQ-15, 3, 5), two slightly favored CXCR4 calcium flux over MAGI-HIV (1, 2) and two favored HIV-MAGI over calcium flux (AMD3100 and 7). The two compounds that favored MB-231 apoptosis (8 and MSX-122) had no clear link to functional CXCR4 response in the calcium flux assay. In all cases where there was measurable Nef-M1 inhibition of 1 μM or below (1–6, 8, IT1t, TIQ-15 and AMD3100) there was measurable HIV-MAGI inhibition that correlated. However, the breast cancer apoptosis had variable correlation to the CXCR4 functional responses pointing to a possible CXCR4-linked secondary mechanism that our assays were not capable of measuring.

It can be appreciated that a given molecule requires certain structural features to provoke or inhibit a given biological response. For instance, the installation of a trifluoromethyl moiety on compound 7 completely abrogates its ability to depolarize MDA-MB-231 cells (EC_50_ = 85 μM) relative to compound 6 which is identical to 7 but bears a hydrogen atom in place of a trifluoromethyl group. This potency loss is also observed for M1 inhibition, where 6 is exceedingly more potent (EC_50_ = 53 nM) than 7 (EC_50_ = 12.7 μM). Compound 5 is similar to 6, however, 5 features an amino butyl side chain that confers a significant gain in potency with respect to M1 inhibition (EC_50_ = 10 nM) and breast cancer depolarization (EC_50_ = 0.39 nM). In fact, compounds 1-5 all feature a flexible alkyl amino side chain which appears to be critical for activity. These compounds inhibit M1-induced apoptosis at low nanomolar and sub-nanomolar concentrations despite other structural variances such as substitutions at the THIQ nitrogen (3, 4). The presence of a methyl-pyrazine stemming from the THIQ nitrogen of Compound 3 confers a 15-fold increase in activity over its methyl-pyrimidine analogue, Compound 4. Compounds 1 and 2 were also more potent than Compound 4 in this assay, indicating that the methylpyrimidine substitution is not the best choice to inhibit M1 from our THIQ and pipirazine series. Although 4 displays mediocre activity against M1 relative to its methylpyrazine analogue compound 3, it is interesting to note that 4 failed to depolarize MDA-MB-231 cells up to 100 μM whereas 3 depolarized half of the cell culture at 15 nM. It is clear that this relatively minor structural permutation between 3 and 4 has a profound effect on their ability to impact the viability of breast cancer cells.

There are several examples of significant consideration. First, is compound 4 an N-THIQ analog of TIQ-15 and has relatively good potency in blocking NefM1 apoptosis (52 nM) but has no effect upon MBA-MD-231 cells up to 100 μM. This great difference can be best explained by the selectivity this compound has for blocking HIV entry (3 nM) over inhibiting the SDF-1/CXCR4 functional response (7,700 nM). Second are compounds 8 and MSX-122 which have no measurable CXCR4 functional response but clearly have potent effects (EC_50_< 100 nM) in the MDA-MB-231 cell line. Compound 8 does have considerable HIV entry blocking ability but in the range of 50-fold higher for the MAGI and NefM1 assays versus the MBA-231 result. While MSX-122 has been reported to have no measurable intrinsic HIV activity or CXCR4 functional response, it does have residual but very weak activity in the NefM1 assay and a potent response against the MBA-MD-231 cells (EC_50_ = 81 nM). In concurrence with previously published observations, this result points to a secondary CXCR4-related mechanism which may be specific to certain cancer cell types such as MDA-231 breast cancer cells.

## DISCUSSION

CXCR4 plays a central role in the pathogenesis of HIV and several cancers and Nef is a small viral protein produced by HIV to subvert the innate and humoral immune response to optimize conditions for viral replication. In 2004, the Bond lab demonstrated that Nef interacts with CXCR4 to dispose of uninfected CD4^+^ T cells which likely contributes to AIDS progression *in vivo* [14, 15]. We saw this as an untapped opportunity to potentially curb CD4 decline by targeting CXCR4 instead of the viral machinery. One advantage of this strategy is that CXCR4 is less prone than the virus to selective pressure, due to its role in cell growth and development. Another advantage of targeting CXCR4 is that it is a co-receptor for viral entry that grants HIV access to a wider distribution of cells and tissues resulting in exacerbated disease. We incubated Jurkat cell cultures with an array of CXCR4 antagonists and co-incubated the cultures with a 10-mer Nef peptide (M1), which is the sequence responsible for Nef's ability to bind CXCR4 and subsequently to initiate the apoptotic program in PBMCs. Our findings reveal that M1-mediated apoptosis in Jurkat cells is readily blocked by our THIQ and piperizine CXCR4 antagonists at therapeutically relevant concentrations (<10 nM). With respect to conventional CXCR4 antagonists previously described, AMD3100 demonstrated unimpressive anti-apoptotic activity against M1 (EC_50_ = 473 nM) when compared to IT1t (EC_50_ = 0.7 nM). MSX-122 is a partial CXCR4 antagonist that fails to displace CXCL12 and weakly mobilizes Ca^2+^ ions upon binding to the receptor [39]. In accord with this precedent, MSX-122 did not readily protect Jurkat cells from M1-induced apoptosis (EC_50_ = 12.0 μM). These findings reveal that CXCR4 antagonism may provide a therapeutic benefit beyond its role in viral entry to prevent the loss of CD4^+^ T cells mediated by Nef during chronic HIV infection. Unsurprisingly, Nef is not the only viral protein produced by HIV-1 that utilizes CXCR4 to eliminate T cells and cause disease. CD4^+^ T lymphocytes experience autophagic cell death upon exposure to HIV-1 envelope glycoproteins (Env) [25]. Glycoprotein 120 (gp120) triggers apoptosis in both CD8^+^ T cells [26] and neuronal cells that leads to the development of HIV-related dementia [40]. Both proteins bind CXCR4 to mediate these effects. Consequently, CXCR4 antagonists also should have the potential to confer protection against Nef, gp120, and Env, as well as preventing viral entry which may prove to be an attractive strategy to curtail the pathogenic nature of HIV-1 in the future.

In the attempt to blockade Nef, we discovered that our CXCR4 antagonists demonstrate profound apoptotic activity against MDA-MB-231 breast cancer cells *in vitro* similar to the effect of Nef M1 peptide. Compound 2 proved to be exceptionally potent (EC_50_ = 370 pm) and is nearly two million-fold more active than AMD3100. In fact, three other compounds in Figure 1 depolarize MDA-MB-231 cells at concentrations <1.0 nM. This phenomenon was selective for CXCR4 positive breast cancer cells and the viability of both Jurkat and CXCR4 negative MDA-MB-468 cells was not compromised upon exposure to high concentrations of any of the antagonists studied (10 μM). Compounds 4 and 6 failed to depolarize MDA-MB-231 cells at concentrations below 10 μM, however, they demonstrated potent inhibition of Nef M1 (EC_50_ ~50 μM) induced apoptosis. Conversely, 8 and MSX-122 weakly inhibit M1-induced apoptosis in Jurkat cells (EC_50_ = 1.10 μM and 12.0 μM, respectively) but are relatively potent initiators of the apoptotic program in MDA-MB-231 cells (EC_50_ = 28 nM and 81 nM, respectively). It's also noteworthy that these compounds did not show any appreciable activity by our CXCL12-calcium flux signaling functional assay. One hypothesis is that these molecules occupy an allosteric site within CXCR4 not previously identified which does not interfere with CXCL12 or M1 signaling, but which may be partially required by CXCR4 and some as yet unidentified ligand to signal the intracellular milieu in breast cancer cells. This mechanism is likely CXCL12-independent based on the assay data. In fact, CXCL12 transfection in MDA-MB-231 cells actually induces apoptosis rather than promoting survival [41]. Further evidence that CXCL12 is not the cognate survival ligand in CXCR4^+^ cancer cells was provided by Dwinell *et al.* who found that CXCL12 is transcriptionally silenced in six of eight breast cancer cell lines including MDA-MB-231 [42]. When CXCL12 expression was reestablished to normal levels, mammary carcinoma cells experienced reduced *in vitro* chemotaxis and *in vivo* metastasis leading to the proposal that CXCL12 silencing promotes mammary neoplastic transformations. Similar to our findings here, Bristol-Meyers Squib (BMS) recently discovered a CXCR4-specific monoclonal antibody (BMS-93654) that induces apoptosis in several CXCR4^+^ tumor models including AML, NHL, and multiple myeloma [43]. Treatment with BMS-93654 resulted in near complete tumor remission and demonstrated similar activity to rituximab. To examine if BMS-93654 prevents CXCL12 binding to CXCR4 to initiate the apoptotic program, the authors subjected the tumors to an anti-CXCL12 antibody instead of BMS-93654. Surprisingly, the anti-CXCL12 antibody failed to suppress tumor growth indicating that inhibition of the CXCL12/CXCR4 axis is not responsible for antitumor activity. When taken together, it is clear that disruption of the CXCL12/CXCR4 axis with small molecule CXCR4 antagonists or a CXCR4-specific antibody is not the mechanism by which CXCR4-dependent tumors undergo apoptosis. Further, it remains unclear as to how Nef (and M1) initiates the apoptotic program through CXCR4 and what downstream signaling cascade is operative.

In summary, we have demonstrated that CXCR4 antagonists protect Jurkat cells against HIV-1 Nef and induce apoptosis in MDA-MB-231 breast cancer cells. Several of our THIQ and piperazine compounds selectively dispose of breast cancer cells at sub-nanomolar potencies which significantly rival the FDA-approved antagonist, AMD3100. This study illuminates the possibility of targeting other HIV proteins to prevent T cell loss and raises interesting questions about the mechanism by which CXCR4 antagonists initiate the apoptotic program in tumors. Furthermore, this study provides the field with a molecular tool set of differing CXCR4 antagonists to study receptor and cellular behavior as it applies to immune system regulation, HIV infection, and cancer pathology.

## MATERIALS AND METHODS

### Cells and cultures

Jurkat cells used in these experiments are CD4+-T-cell lines derived from human T-cell leukemia and human cutaneous T-cell lymphoma cells, respectively, and were obtained from the NIH AIDS Research and Reference Reagent Program. MDA-MB-468, MCF-7, and MDA-MB-231 cells were derived from human breast adenocarcinoma and human breast carcinoma cells, Human umbilical vein endothelial cells (HUVECs), human THP-1 leukemia cells, DU4475, and U937 cells were purchased from American Type Culture Collection (Manassas, VA). These cells were maintained in RPMI 1640 medium supplemented with 2 mM L-glutamine, 4 g/L glucose, 1.0 mM sodium pyruvate, 10 mM HEPES, 100 U of penicillin per ml, 100 U of streptomycin per ml, 25 mM Hepes and 10% fetal bovine serum (Thermo Fisher, Gaithersburg, MD.) at 37°C in 5% CO_2_. For the induction of differentiation to macrophages, the THP-1 monocyte cells (5 × 10^5^ to 10^6^ per ml) were placed in macrophage serum-free medium (macrophage-SFM; Gibco BRL) with 200 nM PMA (phorbol 12-myristate 13-acetate, Millipore Sigma, St. Louis, MO.) for 24 hours. After incubation, nonattached cells were removed by aspiration, and the adherent cells were washed three times with the medium.

### CXCR4 antagonists

The compounds used in this study are shown in Figure 1. AMD3100 was purchased from Sigma-Aldrich as the octahydrochloride salt. All other compounds were synthesized based on previously reported procedures: TIQ15 and 1–4; [35] 5–7; [36] 8; [37] IT1t; [38] MSX-122 [39].

### Total RNA isolation and reverse transcription (RT)-PCR

Total RNA was extracted from Jurkat, THP-1, U937, HUVEC, MDA-MB-468, MDA-MB-231 cells using the RNAzol^TM^ B (TEL-TEST, INC. Friendswood, TX) procedure following the manufacturer's instructions. Five micrograms of RNA were reverse transcribed with SuperScript^TM^ III One-Step RT-PCR System with Platinum Taq High Fidelity (Invitrogen Life Technologies). The following sequences of human CXCR4 primers used for PCR amplification of region 2984 to 4081 of human CXCR4 (GenBank accession no. AF005058) were:

1. hCXCR4-1, 1097 bp of CXCR4 (forward): 5′-ATGAAACTTGGGGCGAGGAC -3′; (reverse): CGGTGTAGTTATCTGAAGTG -3′;
2. hCXCR4-2, 922 bp of CXCR4 (forward): 5′ –ATGTC CATTCCTTTGCCTCT -3′; (reverse): 5′ –AAAGCATAGA GGATGGGGTT -3′;
3. hCXCR4-3, 508 bp of CXCR4 (forward): 5′ –TACCT GGCCATCGTCCACGC -3′; (reverse): 5′ –TCCAAACA CGAGTGCATACC -3′.

The cDNA synthesis and pre-denaturation were 55°C for 30 min and 94°C for 2 min, followed by 35 cycles of PCR amplification, which included denaturation at 94°C for 15 s, annealing at 60°C for 30 s, extend at 68°C for 1 min, and extension at 68°C for 5 min.). PCR products were visualized on 1.5% agarose gels containing ethidium bromide. Gels were photographed using the Fotodyne FOTO/Analyst Luminary Workstations (Fotodyne, Inc., Hartland, WI.) Fluorescence intensity of PCR product bands was quantitated using ImageJ (NIH OpenSource). The PCR products were then purified by QIAquick Gel Extraction Kit (QIAGEN, Valencia, CA.), and confirmed by sequencing using Applied Biosystems 3130xl Genetic Analyzer with Data Collection software V3.0 (Thermo Fisher, Foster city, CA).

### PMA treatment and JC-1 stain

THP-1 cells were grown to 80% confluence in 35 mm of plates, either untreated or treated with PMA (200 nM) for 24 hours or 48 hours and were treated with 100 ng/ml HIV-1 Nef protein or 10 ng/ml of Nef Motif1 for 24 hours. The cells were washed in 1x PBS and centrifuge at 350 × g for 5 min at 4°C. Next, 4 μl of JC-1 reagent (2.5 mg/ml in DMSO) was added into 1 ml) of cell culture medium with 10% Fetal bovine serum at 37°C, which was mixed and immediately added to the cell pellet and incubated for 10 min at 37°C. After incubation, cells were washed with image buffer (137 mM KCL, 3.6 mM NaCl, 0.5 mM MgCl_2_, 1.8 mM CaCl_2_, 1.6 mM NaH_2_PO_4_ and 4.3 mM NaHCo_3_, pH 7.4), and imaged by fluorescence microscopy.

### TUNEL assay

Cells were grown to 80% confluence in 35 mm plates, either untreated or treated with 10 ng/ml of Nef Motif1 or CXCR4 antagonists for 24 hours. The cells were washed twice in ice-cold PBS and then fixed in 4% paraformaldehyde 1 hour. TUNEL assay for apoptosis and microscopy were done as described previously [44].

### Cell viability assay (FD/PI)

A fluorescein diacetate/propidium iodide (FD/PI) viability assay was performed, as previously described [44], on Jurkat cells and THP-1 macrophage cells were treated with 1000 nM AMD3100 or 50 nM TIQ-15 and 50 nM IT1T at 37°C for 24 hours. After 24 hours, the cells were washed with 1X PBS. Five microliters of a 5-mg/ml stock of FD (Sigma) was diluted with 1.25 ml of fresh 1X PBS to make the FD working solution. The FD/PI cocktail was prepared by adding 0.5 ml of the FD working solution to 125 μL of a 20 μg/ml stock of PI (Sigma). A sufficient quantity of FD/PI cocktail was added to resuspend the cells, followed by incubation at room temperature for 3 min. The cells were then transferred to a coverslip and immediately analyzed by epifluorescence at 450-nm excitation and 520-nm barrier, using a computer-controlled Zeiss microscope system (Thornwood, NY). The images were captured with a charged-coupled-device (CCD) camera (MC 100 SPOT, 60910; Photonic Science, East Sussex, United Kingdom) and examined with Image-Pro Plus 2.0 software (Media Cybernetics, Silver Spring, MD).

### MAGI antiviral assay with HIV-1_IIIB_

### Cell preparation

MAGI-CCR5/CXCR4 cells (obtained from the NIH AIDS Research and Reference Reagent Program) are passaged in T-75 flasks prior to use in the antiviral assay. MAGI-CCR5/CXCR4 cells are derived from HeLa-CD4-LTR-β-gal cells. The cells have been engineered to express high levels of CD4 and CXCR4 and contain one copy of the HIV-1 LTR promoter driving expression of the β-galactosidase gene upon HIV-1 Tat transactivation. On the day preceding the assay, the cells are plated at 1 × 10^4^ well and incubated at 37°C overnight. Total cell and viability quantification is performed using a hemacytometer and trypan blue exclusion. Cell viability is greater than 95% for the cells to be utilized in the assay.

### Virus preparation

The virus used for these tests is the CXCR4-tropic strain HIV-1IIIB. This virus was obtained from the NIH AIDS Research and Reference Reagent Program and was grown in Ghost Hi5/MAGI-CCR5/CXCR4 co-cultures for the production of stock virus pools. For each assay, a pre-titered aliquot of virus is removed from the freezer (−80°C) and allowed to thaw slowly to room temperature in a biological safety cabinet. The virus is re-suspended and diluted into tissue culture medium such that the amount of virus added to each well in a volume of 50 μL is approximately ten TCID50/well (~0.001 TCID50/cell).

### Assay setup

Compounds are evaluated at one or two concentrations (e.g., for initial screening) or in dose-response at six concentrations (triplicate wells/concentration). On the day of assay setup, compound dilutions are prepared at two-times (2×) the final required concentrations. Media used for plating the cells the day before assay setup is aspirated from the plates and replaced with 50 μL of the 2× compounds, followed by the addition of 50 μL of virus, which dilutes the compounds to the final 1X concentrations. Cell control wells (cells only) and virus control wells (cells plus virus) are included on each assay plate. Identical uninfected assay plates (virus replaced with media) are prepared for parallel cytotoxicity testing. The cultures are incubated for 48 hours or 6 days (depending on compound or client requirements) after which antiviral efficacy is measured as the inhibition of β-galactosidase reporter expression and cytotoxicity is monitored by MTS staining.

### β-galactosidase chemiluminescent endpoint analysis

A chemiluminescent endpoint is used to determine the extent of β-galactosidase expression as a measure of HIV-1 infection of the cells. Once HIV-1 has attached and entered the MAGI-CXCR4 cells, HIV-1 Tat transactivates the LTR dependent β-galactosidase enzyme to express higher than normal levels of β-galactosidase. Thus, there is a direct relationship between the level of HIV-1 infection and the level of β-galactosidase detected in the cells. At 48 hours or 6 days post infection, plates are aspirated, and PBS is added to each well. Gal-screen reagent (Tropix, Bedford, MA) is then added per the manufacturer's instructions for chemiluminescent detection of β-galactosidase activity and incubated at room temperature for 90 minutes. The resulting chemiluminescence signal is then read using a Microbeta Trilux luminescence reader (PerkinElmer/Wallac).

### MTS staining for cell viability

At assay termination, the cytotoxicity assay plates are stained with the soluble tetrazolium-based dye MTS (CellTiter Reagent, Promega) to determine cell viability and quantify compound toxicity. MTS is metabolized by the mitochondrial enzymes of metabolically active cells to yield a soluble formazan product, allowing the rapid quantitative analysis of cell viability and compound cytotoxicity. The MTS is a stable solution that does not require preparation before use. At termination of the assay, 15 μL of MTS reagent is added per well. The microtiter plates are then incubated at 37°C for 1.5–2 hrs. The incubation interval was chosen based on empirically determined times for optimal dye reduction. The plates are read spectrophotometrically at 490/650 nm with a Molecular Devices V_max_ plate reader.

### Data analysis

Percent inhibition of virus replication and percent cell viability at each concentration are calculated using an in-house computer program. For dose-response testing, IC50 (50% inhibition of virus replication), IC90 (90% inhibition of virus replication), TC50 (50% cytotoxicity), and therapeutic index values (TI = TC50/IC50; also referred to as Antiviral Index or AI) are provided. Raw data for both antiviral activity and cytotoxicity with analyzed/tabulated data are provided in a printout summarizing the individual compound activity. For dose-response testing, a graphical representation of the data is also provided. An IC_50_ of 2 to 5 nM was obtained for AMD3100.

### Calcium flux assay

Calcium flux in human CXCR4–expressing (full length cDNA) Chem-1 cell line induced by SDF-1α. CXCR4 –expressing Chem-1 cells were loaded with Fluo-4 and calcium flux in response to recombinant human SDF-1α (10^−5^ to 10^−9^ M) was determined in triplicate on a Molecular Devices FLIPR-TETRA™ Flex Station. Inhibition of SDF-1α-mediated calcium flux in CXCR4-expressing CEM cells by test compounds. CXCR4- expressing CEM cells were loaded with Fluo-4, washed, and pre- incubated with the indicated concentrations of test compounds for 10 min. Calcium flux in response to 5 nM recombinant human SDF-1α was determined on a Molecular Devices Flex Station. An EC_50_ for calcium mobilization by SDF-1α of ~4 nM with Signal/noise at ligand E_max_= 542 Z' = 0.82 with SDF-1α at the EC_50_. An IC_50_ of 57 nM was obtained for AMD3100.

### CXCR4 surface expression assay

Cell surface CXCR4 expression was determined in various cell types. Cells were incubated with medium at 37°C for 48 hours. Cells were collected and centrifuged at 350 × g at 4°C for 5 minutes, washed by PBS and resuspend cell pellet in 1% BSA, and incubated at 4°C for 15 minutes. After incubation, cells were centrifugated, the pellet were added appropriately PE/anti-human CXCR4 antibody in Cell Staining Buffer (BioLegend) and incubated at 4°C for 1 hour in the dark and then washed with PBS. The cell surface CXCR4 expression was determined on MDF-7, MDA-MB-231, MDA-MB-468, MDA-MB-468 (knock-in CXCR4) breast cancer cells, non-tumorigenic MCF-10A cells, HUVEC primary cells, and THP-1 monocytes and Jurkat lymphocytes via Flow Cytometry.

### CXCR4 knock-in MDA-MB-468 cells

MDA-MB-468 cells were transfected with 2.5 μg of pcDNA3.1/Zeo (+) CXCR4 via a electroporator (BIO-RAD), which was set at 250 V (0.25 KV) and 960 uF, and then cells were incubated at 37°C for 24 hours. Cells were treated with CXCR4 compounds either MDA-MB-468 knock-in CXCR4 cells or non-CXCR4 MDA-MB-468 cells at 37°C for 24 hours and following by JC-1 satin and detected by Fluoresce Microscopy.
